# Impact of root canal treatment on oral health-related quality of life: a prospective cohort study

**DOI:** 10.1590/1807-3107bor-2025.vol39.126

**Published:** 2025-12-01

**Authors:** Ludmila Silva GUIMARÃES, Erlange Andrade Borges da SILVA, Fernanda Garcias HESPANHOL, Marcelo Levin Cidade D’Amato TAVARES, Lívia Azeredo Alves ANTUNES, Leonardo Santos ANTUNES

**Affiliations:** (a) Universidade Federal Fluminense – UFF, School of Dentistry,Postgraduate Program, Niterói, RJ, Brazil.; (b) Universidade Federal Fluminense – UFF, Health Institute of Nova Friburgo, Department of Specific Formation, Nova Friburgo, RJ, Brazil.; (c) Universidade Federal Fluminense – UFF, School of Dentistry, Department of Specific Formation, Nova Friburgo, RJ, Brazil.

**Keywords:** Periapical Periodontitis, Oral Health, Quality of Life, Root Canal Preparation

## Abstract

The objective of this study was to assess the impact of root canal treatment (RCT) in single-rooted teeth with asymptomatic apical periodontitis on oral health-related quality of life (OHRQoL). As a secondary objective, the impact of factors such as age, gender, ethnicity, tooth groups, arch position, crown destruction, postoperative pain, edema, and use of analgesics after procedure were also noted. A prospective cohort study was designed and enrolled in a sample of 105 patients who needed root canal treatment in single-rooted maxillary or mandibular teeth with asymptomatic apical periodontitis, without preoperative symptoms. RCT was performed in one session. The impact of the OHRQoL was recorded using the Oral Health Impact Profile (OHIP-14) at baseline and after seven and 30 days. Predictor variables were also collected: age, gender, ethnicity, tooth groups, arch position, destruction of the crown, postoperative pain, edema, and the use of analgesics. Data were tabulated and analyzed in SPSS software, with alpha value set at 0.05. All domains of the OHIP-14 questionnaire revealed a statistically significant difference between the times evaluated (p < 0.001), showing the positive impact of the RCT on OHRQoL, with moderate and large effects. There was a significant difference in the total score for gender (p = 0.001), ethnicity (p = 0.010), and crown destruction (p = 0.002). RCT improved OHRQoL scores in all domains assessed by OHIP-14, with moderate and large effects. Factors such as female gender, mixed race and afrodescendant participants, and extensive crown destruction negatively influenced the OHRQoL of patients pre-RCT.

## Introduction

Daily activities such as eating, talking, and smiling are crucial to an individual’s well-being. Therefore, it is now understood that oral health is an integral part of general health.^1^ In endodontics, there is currently a paradigm shift, with treatment needs and outcomes also being assessed from the patient’s perspective^2^ rather than relying only on the clinician’s viewpoint. Results based on the patient’s self-perception of oral health and oral health-related quality of life (OHRQoL) provide an important opportunity to complement clinical data with the views of patients, and are significant in clinical dental practice, dental education, and research, allowing for better clinical decision-making.^1,3^

Different approaches to measuring OHRQoL have been developed over time and vary in terms of the number of questions or areas being investigated.^4^ The short-form Oral Health Impact Profile (OHIP) is the most utilized and is a well-established, standardized measure that has been adapted for use in several languages such as Brazilian Portuguese.^5^

The prevalence of teeth with clinical evidence of pulp and/or periapical tissue problems in patients is high.^6^ It is widely known that oral conditions, including endodontic issues, can have varying effects on daily life.^1^ Systematic reviews have shown that root canal treatment can positively impact OHRQoL, but these reviews have also concluded that the lack of well-designed studies undermines the strength of recommendation of this evidence.^7,8^ In fact, knowledge about the impact of root canal treatment on OHRQoL^8^ and key factors associated^2,9-14^ with its impact remains limited.

Therefore, the main objective of this study was to assess the impact of root canal treatment in single-rooted teeth with asymptomatic apical periodontitis on OHRQoL. The null hypothesis tested was that root canal treatment may not influence OHRQoL. In addition, as a secondary objective, the impact of factors such as age, gender, ethnicity, tooth groups, arch position, crown destruction, postoperative pain, edema, and use of analgesics after procedure were also evaluated.

## Methods

### Study design

This prospective cohort study was approved by the Research Ethics Committee of Universidade Federal Fluminense/Health Institute of Nova Friburgo (No. 2.353.996) and was reported according to the Guidelines for reporting non-randomized studies^15^. All research participants signed the informed consent form and were informed of the risks and benefits of the treatment.

### Patient Selection

The sample size was determined using the G*Power software (version 3.1). It was expected to achieve an intermediate effect size (Cohen > 0.5) (psychometrica.de/effect _size). The mean baseline OHIP-14 score of 15.1 (standard deviation [SD] = 10.2) and the mean one-month post-treatment score of 8.9 (SD = 8.6) were used, according to Liu et al.^12^, obtaining an effect size of 0.657. Considering an alpha error of 0.05, and a beta error (power) of 0.9, 105 participants were required.

Patients presenting to the Faculty of Dentistry of the Universidade Federal Fluminense/Health Institute of Nova Friburgo, Rio de Janeiro, Brazil, with deep caries lesions, without pulp exposure, and/or dental trauma, between March 2019 and February 2020, were evaluated.

Individuals older than 18 years who needed at least one root canal treatment in single-rooted teeth on the maxilla or mandible with necrotic pulp and asymptomatic apical periodontitis were included. The status of the pulp was determined by cold and heat sensitivity tests. In addition, the diagnosis of apical periodontitis was also confirmed, through digital radiographs (Kodak, Rochester, USA).

The exclusion criteria were as follows: patients with preoperative pain/oedema as these are aetiological factors associated with flare-ups; pregnancy; lactation; allergy to sodium hypochlorite (NaOCl); or the drug ibuprofen; and patients who used antibiotics in the last 30 days^14^ and analgesics/anti-inflammatories 24 hours prior or requiring antibiotic pre-medication for dental treatment. Teeth wherein foramen patency cannot be determined, cases of root canal retreatment, and vital teeth or teeth with necrosis and without apical periodontitis were also excluded.

### Treatment protocol

Clinical procedures were performed by a single specialist (L.S.G) with 10 years of experience in a single session, using a standardized protocol.

The research participants were anaesthetized with 2% lidocaine with 1:100,000 epinephrine (DFL Indústria e Comércio Ltda, Taquara, Brazil), and endodontic access was performed with a sterile diamond bur (KG Sorensen, Cotia, Brazil) at high speed. Rubber dam was placed and disinfected with 2.5% NaOCl (Fórmula & Ação, São Paulo, Brazil). Each tooth was irrigated with the same volume of irrigant and 15 mL of 2.5% NaOCl (Fórmula & Ação, São Paulo, Brazil).^16^

The working length (WL) determination was performed through the apical locator RomiApex A-15 (Romidan, Kiryat Ono, Israel), at the “00” mark,^14,17^ and the patency maintained with a size 10 K-file (Dentsply Sirona, York, USA). Files 40 or 50 of the Reciproc system (VDW, Munich, Germany), coupled to the VDW Silver engine (VDW, München, Germany), were selected for the shaping of root canals. In cases where a size 30 K-file did not move passively to the WL, R40 was selected. In cases where a size 30 K-file passed passively to the WL, R50 was selected, according to the manufacturer’s protocol. The instruments were introduced with linear back-and-forth movements with an amplitude of 2-3 mm and slight apical pressure. The files were discarded after a single use.

Lastly, final irrigation with 5 mL of 17% EDTA for 5 min,^17,18^ neutralization with 2 mL of saline solution, drying of the root canal with sterile absorbent paper cones from the Reciproc system (VDW, Munich, Germany), and root canal filling with gutta-percha of the system with MTA Fillapex sealer (Angelus, Londrina, Brazil), using lateral condensation, was performed. A definitive restoration was performed, and the occlusion was checked and adjusted.

### Outcome variable

The analysis of the impact of the OHRQoL of patients was recorded using the short-form Oral Health Impact Profile (OHIP-14). This questionnaire is composed of 14 items conceptually distributed in seven dimensions (functional limitation, physical pain, psychological discomfort, physical disability, psychological disability, social disability, and handicap). Each dimension is evaluated by two items and scored on a five-point Likert scale as follows: 0 = never; 1 = hardly ever; 2 = occasionally; 3 = fairly often; and 4 = very often. OHIP-14 scores were calculated using the additive method, which can vary between 0 and 56, with higher scores indicating poorer OHRQoL.^5^.In the present study, OHIP-14 was applied, through face-to-face interviews, at three different times: before root canal treatment with foraminal enlargement (T0); after seven days (T7); and after 30 days (T30) by an evaluator, who did not participate in the clinical procedure (E.A.B.S), in a private room at the dental clinic.

### Predictor variables

Data on the following predictor variables were collected and related to the OHRQoL outcome variables: age; gender (male or female); ethnicity (caucasian/ mixed race/ afrodescendant); tooth groups (anterior or posterior); arch position (maxilla or mandible); and destruction of the crown, which was clinically evaluated as small (less than 1/3 coronary destruction) or extensive (greater than 1/3).

Postoperative pain, edema, and use of analgesics after root canal treatment in single-rooted teeth with asymptomatic apical periodontitis were also noted. The postoperative pain analysis was performed using a visual analogue scale (VAS), which was delivered to all patients to record the pain assessment on the 1st, 2nd, 3rd, 4th, 5th, 6th, 7th, 30th after root canal treatment. Edema was evaluated at 48 h, 72 h, and 7 days after treatment, based on the criteria of Morse et al.^19^. Two independent and blinded authors clinically evaluated oedema by comparing the patient’s initial photograph (k = 0.90), as follows:

- Mild: no distortion of the face but presents slight swelling of the gingiva, cheeks or chin;
- Moderate: superficial distortion of the cheek or chin, and;
- Severe: severe distortion of the part involved.

All patients were instructed to contact the research coordinator (L.S.A) in case of pain or any other complication. In cases of pain, the ibuprofen 400 mg, every 6 hours for 5 days, was prescribed^14,20^, according to the pre-established protocol.

### Statistical analysis

The normality of the data was verified using the Shapiro-Wilk test. The Wilcoxon test was performed to compare the OHRQoL at T0 vs. T7 and T0 vs. T30, and Mann-Whitney or Kruskal-Wallis tests, with post-hoc Dunn’s test, were used to compare the OHRQoL scores per domain between groups at T0. Multivariate conventional linear regression was performed to evaluate the impact of age, gender, ethnicity, and destruction of the crown on the OHRQoL before root canal treatment (T0). Multivariate linear regression using the generalized estimating equation model (GEE) was also performed to evaluate the impact of the predictor variables and of the variables obtained during follow-up (postoperative pain, edema, and the use of analgesics) on the OHRQoL after the root canal treatment (T0 vs. T7 and T0 vs. T30).

IBM SPSS version 25.0 (IBM Corp, Armonk, New York, USA) was used for all analyses, and the alpha value was set at 0.05. The standardized response mean (SRM) and effect size (ES) were calculated using MedCalc (MedCalc Software Ltd, Ostend, Belgium). SRM was calculated to test the responsiveness to change over time by obtaining the ratio of the observed change in OHIP-14 scores to the standard deviation of changes^21^. A value of 0.5 denoted null responsiveness and 1.0 denoted perfect responsiveness^22^. ES values were classified as follows: ≤ 0.2, small effect; 0.3–0.7, moderate effect; and ≥ 0.8, large effect.^23^

## Results

One hundred and five patients were included in this study, and no patients were lost during follow-up (Figure). Each patient contributed only with 1 tooth. The mean age of the sample was 40.32 (SD = 13.35) (range 18–70 years), and the majority were female (61.0%) and caucasian (54.5%). Anterior teeth (83.8%), positioned in the maxilla (63.8%), and with small crown destruction (59.0%) were the most prevalent variables.

**Figure:**
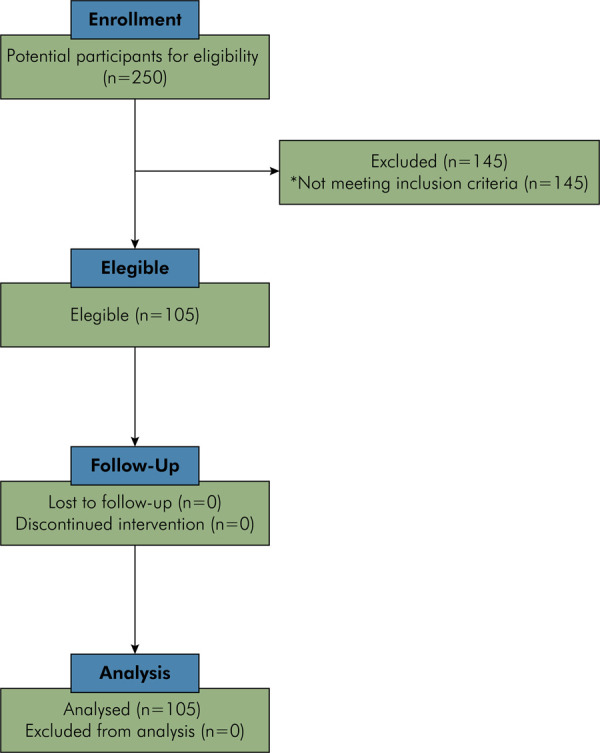
Flow diagram of the sample.

Table 1 presents the mean comparisons of the OHIP-14 domains according to the evaluation time. All domains of the OHIP-14 questionnaire showed a statistically significant difference between the times evaluated (T0 vs T7 / T0 vs T30) (p < 0.001), showing the positive impact of the root canal treatment on OHRQoL. In comparing between the T0 and T7 intervals, SRM values were greater than 0.5 only in the psychological discomfort domain, representing perfect responsiveness. Large effect sizes were observed in the overall score and in physical pain, psychological discomfort, and psychological disability, at both times evaluated. The other variables had a moderate effect size.

**Table 1:** Mean comparisons between times per domain. n(%)

Domains	T0 Mean (SD)	T7 Mean (SD)	Mean of difference (SD)	p-value	ES	SRM	T30 Mean (SD)	Mean of difference (SD)	p-value*	ES	SRM
Functional limitation	1.09 (1.79)	0.12 (0.56)	0.96 (2.58)	< 0.001	0.72	0.37	0.07 (0.39)	1.01 (2.73)	< 0.001	0.56	0.37
Physical pain	3.10 (2.21)	1.13 (1.92)	1.97 (4.84)	< 0.001	0.89	0.40	0.56 (1.32)	2.54 (5.62)	< 0.001	1.14	0.45
Psychological discomfort	4.46 (2.52)	1.10 (1.74)	3.36 (2.76)	< 0.001	1.33	1.21	0.74 (1.37)	3.71 (7.92)	< 0.001	1.47	0.46
Physical disability	2.03 (2.33)	0.56 (1.41)	1.46 (3.79)	< 0.001	0.62	0.38	0.18 (0.63)	1.84 (4.38)	< 0.001	0.79	0.42
Psychological disability	3.34 (2.44)	0.62 (1.57)	2.72 (6.08)	< 0.001	1.11	0.44	0.36 (0.97)	2.98 (6.49)	< 0.001	1.21	0.45
Social disability	1.44 (2.13)	0.35 (1.24)	1.08 (3.07)	< 0.001	0.50	0.35	0.29 (1.08)	1.15 (3.25)	< 0.001	0.53	0.35
Handicap	1.78 (2.29)	0.22 (0.90)	1.56 (3.85)	< 0.001	0.68	0.40	0.10 (0.65)	1.68 (4.09)	< 0.001	0.73	0.41
Total Score	17.29 (11.32)	4.11 (6.95)	13.17 (28.79)	< 0.001	1.16	0.40	2.33 (4.52)	14.95 (32.15)	< 0.001	1.32	0.46

ES: Effect size; SRM: Standardized response main. *In comparison with T0. Wilcoxon test was performed; bold forms mean statically difference.

The mean scores of the OHIP-14 domains in relation to the predictor variables of gender, ethnicity, tooth groups, arch position, and destruction of the crown were compared at T0 (Table 2). There was a significant difference in the total score for gender (p = 0.001), ethnicity (p = 0.010), and crown destruction (p = 0.002). Women, mixed race and afrodescendant individuals, and patients with extensive destruction of the tooth had higher scores and poorer OHRQoL. Gender showed no association with OHRQoL only in the handicap domain (p = 0.858). Ethnicity was significantly associated with psychological discomfort (p = 0.013). Patients with teeth positioned in the maxilla (p = 0.045) with extensive destruction of the crown (p = 0.006) showed a significant difference in psychological disability and the latter variable in the handicap domain (p=0.009). The tooth groups did not influence OHRQoL.

**Table 2:** Comparisons of scores mean between groups. n (%)

Variables	Scores mean per domain at T0 (SD)															
	Functional limitation	p-value	Physical pain	p-value	Psychological discomfort	p-value	Physical disability	p-value	Psychological disability	p-value	Social disability	p-value	Handicap	p-value	Total Score	p-value
Gender																
Male	0.68 (1.54)	0.048	2.27 (2.08)	0.001	3.37 (2.32)	< 0.001	1.27 (2.18)	0.005	2.76 (2.40)	0.050	0.98 (1.94)	0.028	1.76 (2.41)	0.858	13.20 (10.61)	0.001
Female	1.34 (1.91)		3.64 (2.14)		5.16 (2.41)		2.52 (2.30)		3.72 (2.41)		1.73 (2.22)		1.80 (2.23)		19.91 (11.06)	
Ethnicity																
Caucasian	0.82 (1.68)	0.060	2.82 (2.12)	0.341	3.86 (2.56)^a^	0.013	1.65 (2.21)	0.136	3.02 (2.44)	0.365	1.23 (2.01)	0.507	1.25 (1.93)	0.051	14.65 (11.01)^a^	0.010
Mixed race	1.08 (3.29)		3.29 (2.13)		5.58 (2.04)^b^		2.17 (2.29)		3.75 (2.23)		1.83 (2.22)		2.42 (2.22)		20.12 (10.28)^b^	
Afrodescendant	1.71 (1.94)		3.58 (2.48)		4.75 (2.50)		2.79 (2.51)		3.71 (2.62)		1.54 (2.35)		2.42 (2.87)		20.71 (11.87)^b^	
Tooth groups																
Anterior	1.05 (1.74)	0.616	3.11 (2.23)	0.965	4.65 (2.49)	0.075	1.94 (2.28)	0.420	3.38 (2.46)	0.799	1.48 (2.13)	0.533	1.89 (2.32)	0.334	17.49 (11.07)	0.611
Posterior	1.29 (2.11)		3.06 (2.16)		3.47 (2.52)		2.47 (2.57)		3.18 (2.43)		1.24 (2.22)		1.24 (2.10)		16.24 (12.88)	
Arch position																
Maxilla	1.18 (1.80)	0.447	3.04 (2.32)	0.606	4.61 (2.40)	0.426	1.91 (2.26)	0.493	3.72 (2.52)	0.045	1.64 (2.28)	0.411	1.97 (2.38)	0.301	18.07 (11.32)	0.299
Mandible	0.92 (1.79)		3.21 (2.02)		4.18 (2.73)		2.24 (2.46)		2.68 (2.18)		1.08 (1.82)		1.45 (2.12)		15.89 (11.34)	
Destruction of the crown																
Small	0.92 (1.66)	0.204	3.05 (2.26)	0.587	4.05 (2.48)	0.073	1.71 (2.16)	0.102	2.79 (2.37)	0.006	1.13 (1.84)	0.172	1.32 (2.04)	0.009	14.97 (10.88)	0.002
Extensive	1.36 (1.98)		3.21 (2.18)		5.00 (2.49)		2.55 (2.50)		4.17 (2.36)		1.88 (2.49)		2.48 (2.51)		20.76 (11.33)	

Mann-Whitney test was performed, except to Ethnicity, which was compared by Kruskal-Wallis test with Dun pos-hoc test. The superscript letters indicate the means statistically different. Bold forms mean statically difference.

Several variables were evaluated per domain at T0 using generalized linear regression (Table 3). In the total score, the variables female (p = 0.009), mixed race individuals (p = 0.012), and patients with extensive crown destruction (p=0.028) negatively impacted OHRQoL. However, older age showed an improvement in the OHRQoL in the physical pain domain (r = -0.037; p = 0.047). More details of specific domains are shown in Table 3.

**Table 3:** Generalized Linear Regression per domain at T0.

Domains	Variables	Reference	Factor	p-value	Coefficient	Standard error
Functional limitation	Gender	Male	Female	**0.024**	0.861	0.381
Physical pain	Age*	-	-	**0.047**	-0.037	0.018
Psychological discomfort	Gender	Male	Female	**0.018**	1.100	0.465
	Ethnicity	Caucasian	Mixed race	**< 0.001**	2.100	0.523
			Afrodescendant	0.070	1.162	0.642
	Destruction of the crown	Small	Extensive	**0.001**	1.591	0.469
Physical disability	Gender	Male	Female	**0.003**	1.526	0.504
Psychological disability	Gender	Male	Female	**0.017**	1.187	0.495
	Destruction of the crown	Small	Extensive	**0.011**	1.324	0.523
Handicap	Destruction of the crown	Small	Extensive	**0.023**	1.220	0.538
Total Score	Gender	Male	Female	**0.009**	6.259	2.397
	Ethnicity	Caucasian	Mixed race	**0.012**	6.750	2.691
			Afrodescendant	0.610	1.818	3.568
	Destruction of the crown	Small	Extensive	**0.028**	5.455	2.483

*Means a continuous variable. All characteristics of population were evaluated in a single model, and were related only the significant variables. Bold forms mean statically difference.

Table 4 evaluates the significant variables per domain at T0 vs. T7 using multivariate linear regression using the generalized estimating equation model (GEE). In the total score, teeth positioned in the maxilla (r = 1.301; p < 0.001) with extensive crown destruction (r = 5.406; p < 0.001) and patients with pain during follow-up (r = 2.663; p < 0.001) also negatively impacted OHRQoL. More details of specific domains are shown in Table 4.

**Table 4:** Multivariate Linear Regression by Generalized Estimating Equation Model (GEE) (T0 vs. T7).

Variables	Reference	Factor	p-value	Coefficient	Standard error
Functional limitation					
Gender	Male	Female	**0.031**	0.545	0.251
Ethnicity	Caucasian	Mixed race	**0.001**	0.310	0.091
		Afrodescendant	0.051	0.449	0.230
Arch position	Mandible	Maxilla	**< 0.001**	0.208	0.027
Destruction of the crown	Small	Extensive	**< 0.001**	0.250	0.026
Physical pain					
Gender	Male	Female	**< 0.001**	0.679	0.099
Arch position	Maxilla	Mandible	**0.013**	0.444	0.177
Destruction of the crown	Small	Extensive	**< 0.001**	0.269	0.059
Pain during follow up*^#^	-	-	**< 0.001**	0.711	0.068
Psychological discomfort					
Tooth groups	Posterior	Anterior	**< 0.001**	0.993	0.136
Destruction of the crown	Small	Extensive	**< 0.001**	1.337	0.364
Pain during follow-up*^#^	-	-	**< 0.001**	0.615	0.146
Physical disability					
Edema	No	Yes	**< 0.001**	1.191	0.247
Destruction of the crown	Small	Extensive	**< 0.001**	0.638	0.015
Age*	-	-	**< 0.001**	0.007	0.001
Pain during follow-up*^#^	-	-	**< 0.001**	0.322	0.068
Psychological disability					
Arch position	Mandible	Maxilla	**0.002**	0.723	0.233
Destruction of the crown	Small	Extensive	**< 0.001**	1.156	0.132
Pain during follow-up*^#^	-	-	**< 0.001**	0.267	0.059
Social disability					
Arch position	Mandible	Maxilla	**< 0.001**	0.743	0.177
Ethnicity	Caucasian	Mixed race	**0.019**	0.495	0.210
		Afrodescendant	**< 0.001**	0.118	0.020
Destruction of the crown	Small	Extensive	**< 0.001**	0.877	0.023
Age*	-	-	**0.031**	0.009	0.004
Pain during follow-up*^#^	-	-	**< 0.001**	0.348	0.082
Handicap					
Arch position	Mandible	Maxilla	**< 0.001**	0.179	0.022
Tooth groups	Posterior	Anterior	**< 0.001**	0.618	0.139
Edema	Yes	No	**< 0.001**	0.243	0.025
Destruction of the crown	Small	Extensive	**0.007**	0.893	0.331
Age*	-	-	**< 0.001**	0.006	0.000
Total score					
Arch position	Mandible	Maxilla	**< 0.001**	1.301	0.145
Destruction of the crown	Small	Extensive	**< 0.001**	5.406	0.742
Pain during follow-up*^#^	-	-	**< 0.001**	2.663	0.667

*Means a continuous variable. #The highest score during the 7 days of evaluation was used. All characteristics of population and data obtained during follow up (pain, edema, use of analgesic) were evaluated in a single model, and were related only the significant variables. Bold forms mean statically difference.

When evaluating these variables in T0 vs. T30 using multivariate linear regression by GEE, in total score, teeth with extensive crown destruction (r = 4.310; p < 0.001), pain during follow-up (r = 2.676; p < 0.001), and absence of edema (r = 0.641; p = 0.031) were identified as factors associated with worsening of the OHRQoL. Older age showed an improvement in the OHRQoL, but this time in the psychological discomfort domain (r = -0.019; p = 0.023). More details on the specific domains are shown in Table 5.

**Table 5:** Multivariate Linear Regression by Generalized Estimating Equation Model (GEE) (T0 vs. T30).

Variables	Reference	Factor	p-value	Coefficient (%)	Standard error
Functional limitation					
Arch position	Mandible	Maxilla	< 0.001	0.259	0.008
Pain during follow-up*^#^	-	-	0.040	0.248	0.120
Destruction of the crown	Small	Extensive	< 0.001	0.177	0.024
Physical pain					
Gender	Male	Female	0.014	0.472	0.192
Pain during follow-up*^#^	-	-	< 0.001	0.608	0.012
Arch position	Maxilla	Mandible	< 0.001	0.600	0.006
Destruction of the crown	Small	Extensive	< 0.001	0.235	0.035
Psychological discomfort					
Age*	-	-	0.023	-0.019	0.008
Tooth groups	Posterior	Anterior	< 0.001	0.976	0.147
Physical disability					
Pain during follow-up*^#^	-	-	< 0.001	0.313	0.074
Physical disability					
Destruction of the crown	Small	Extensive	0.009	0.873	0.332
Pain during follow-up*^#^	-	-	< 0.001	0.158	0.017
Social disability					
Pain during follow-up*^#^	-	-	< 0.001	0.418	0.032
Destruction of the crown	Small	Extensive	< 0.001	0.662	0.128
Age*	-	-	< 0.001	0.005	0.001
Handicap					
Edema	Yes	No	< 0.001	0.227	0.036
Destruction of the crown	Small	Extensive	0.011	0.874	0.344
Tooth groups	Posterior	Anterior	< 0.001	0.621	0.137
Age*	-	-	< 0.001	0.003	0.001
Pain during follow-up*^#^	-	-	0.001	0.346	0.104
Total score					
Pain during follow-up*^#^	-	-	< 0.001	2.676	0.658
Destruction of the crown	Small	Extensive	< 0.001	4.310	1.517
Edema	Yes	No	0.031	0.641	0.296

*Means a continuous variable. #The highest score during the 7 days of evaluation was used. All characteristics of population and data obtained during follow up (pain, edema, use of analgesic) were evaluated in a single model, and were related only the significant variables. Bold forms mean statically difference.

## Discussion

Root canal treatment can positively improve the impact on OHRQoL; therefore, the null hypothesis tested was rejected.

The OHIP-14 is used to measure the impact of oral health on quality of life, and analyses seven dimensions more concisely (functional limitation, physical pain, psychological discomfort, physical disability, psychological disability, social disability, and handicap)^24^ in adults. A study conducted by Yaylali et al.^14^evaluated the impact of OHRQoL; however, they used a quality of life scale (QOLS), which is a measure of health-related quality of life. This instrument is not the best method for detecting the impact on oral health due to its non-specificity. The QOLS is a reliable and valid instrument for measuring quality of life from the patient’s perspective. However, it focuses on domains that come from the qualitative descriptions of a wide range of adults across gender, culture, and language groups. It is used with confidence in chronic illness groups such as rheumatic diseases, fibromyalgia, chronic obstructive pulmonary disease, gastrointestinal disorders, cardiac disease, spinal cord injury, psoriasis, urinary stress incontinence, posttraumatic stress disorder, and diabetes.^25^

Therefore, the Brazilian version of the OHIP-14 form was used to assess the impact of the proposed treatment on OHRQoL. Although the OHIP-14 form was not developed to assess OHRQoL changes after root canal treatment, previous studies have confirmed that it is reliable, valid, and responsive for detecting OHRQoL changes after this procedure.^2,9,10,12^ The application of the OHIP-14 in the interview format did not compromise these results, as the psychometric properties of this instrument are not related to the method of administration.^26^ It is also important to point out that recalls are indispensable in the assessment of OHRQoL. The use of a shorter reference period, such as one month, does not appear to influence responses,^27^ in addition to reducing memory bias^2^ Another reason for applying a short recall period is that the treatment effects can be assessed in the short term. This is very important considering that several dental treatments, such as reducing or eliminating pain, have an immediate effect on oral health. However, when administering pre- and post-intervention questionnaires with only a short period between assessments (e.g., one week), the effects of the retest can confound the results.^28^

Data regarding postoperative pain, edema, and use of analgesics after root canal treatment in single-rooted teeth with asymptomatic apical periodontitis were noted and related to OHRQoL. This study included only asymptomatic patients because the inclusion of teeth with preoperative symptoms could influence the results on the visual analogue scale as they are an aetiological factor associated with interappointment flare-ups,^29,30^ and were correlated with a higher OHIP-14 score.^11^ Endodontic postoperative pain can range from 3% to 58%,^31^ and these symptoms are well-known complications in daily clinical practice, which can affect both patients and dentists.^32^ However, the prevalence of pain after root canal treatment is expected, and the severity of pain decreases substantially within the initial days.^33^ The present results showed a worsening of the OHRQoL when this item was evaluated, reaffirming that pain can negatively affect quality of life.^2^ The regression model also indicated that patients who did not present with edema had a worse OHRQoL in the handicap domain. This was probably due to a type I error, in which a statistically significant result is reached when it actually happened by chance. The results of a clinical trial may be subject to random errors due to the variability in the measured data that arises purely by chance^34^. Although the sample size was determined through adequate sample calculation and the use of a validated instrument with the ability to assess changes over time, this random error occurred. The instrument’s ability to detect changes over time in quality of life, or responsiveness, is a property used primarily in clinical trials to test changes during treatment^3^. In this study, the instrument demonstrated perfect responsiveness only in the psychological discomfort domain in the T0 vs. T7 interval. Large and moderate effect sizes were also identified, and this result reflects an important clinical extrapolation, an interesting magnitude of effect in the evaluated domains, signalling that these points really show the patients’ perceptions and changes in their OHRQoL.

In addition, new data on important factors that can influence the quality of life through patient perceptions were scored. In the present study, variables such as age, gender, ethnicity, tooth groups, arch position, and destruction of the crown were evaluated. Of these, female gender, mixed race and afrodescendant participants, maxillary teeth, and extensive destruction of the crown negatively influenced the OHRQoL. Women reported a higher impact of treatment on OHRQoL, in agreement with the study by Khoo et al.^11^. Normally, women report more severe pain levels, more frequent pain, and longer duration of pain than men. Furthermore, women tend to perceive the negative physical, psychological,^35^ and social impacts in relation to their oral health.^36^ In relation to ethnicity, mixed race and afrodescendant patients showed poorer OHRQoL. It is already known that minority groups have a worse quality of life, and the influence of race on oral health perceptions is linked to biological, socioeconomic, behavioural, and psychosocial factors that vary across racial/ethnic groups.^37^ Historically, some groups have experienced greater social exclusion due to racism and discrimination. This may explain their predisposition to present the worst health outcomes.^38^ Finally, maxillary teeth and extensive destruction of the crown also negatively impacted the OHRQoL. These factors demonstrated problems in the psychological disability (difficulty in relaxing and feeling embarrassed) and handicap (feeling that life in general was less satisfactory) domains.^11^ Importantly, minor differences in dental aesthetics, especially in more visible teeth such as positioned in the maxilla, can have a significant effect on the perception of OHRQoL,^39^ reflecting the patient’s difficulty in feeling good about themselves and with society. In the present study, all endodontically treated teeth were rehabilitated, which may have positively influenced their OHRQoL,^40^ based on both esthetic and functional considerations.

The present findings also suggest that there is no negative influence on OHRQoL in relation to the groups of treated teeth. Khoo et al.^11^ reported that tooth type did not significantly influence OHIP-14 scores; however, Montero et al.^2^ showed that mandibular anterior teeth caused the most functional limitation, psychological discomfort, physical disability, and psychological disability. Future studies should clarify and rigorously address these conflicting findings.

The only factor that improved OHRQoL was age in the physical pain and psychological discomfort domains. This factor showed different behaviours in the other domains, and older age worsened the OHRQoL. The age of the patients included in this study was wide, ranging from 18 to 70 years. The literature shows conflicting results regarding age and OHRQoL.^2,10^ However, Grath et al.^36^ concluded that people aged 16 to 64 years reported that oral health has a greater impact on their quality of life than adults aged 65 years or older, similar to the age group assessed.

This study has multiple strengths that increased its internal validity since it had a short-term prospective design, reducing memory bias and the patient’s perception of the procedure. Root canal treatment was performed by a single specialist dentist (L.S.G), and an instrument already validated in this population was used.^5^ However, it is important to emphasize some limitations. First, although the OHIP-14 has been shown to be a reliable instrument for assessing changes in OHRQoL after root canal treatment,^2,9,10,12^ the low specificity of the form questions in relation to root canal treatment makes it difficult to determine which aspects of the protocol impact each domain of the instrument.^9^ Second, many variables were evaluated, and there might have been a type I error in some factors. Third, there may be a Hawthorne effect in which research participants can actively change their behaviour when they know they are being watched and monitored, leading to an overestimation of the effectiveness of an intervention.

Therefore, this study confirmed that root canal treatment improved OHRQoL scores in all domains assessed by OHIP-14 during the proposed follow-up. The magnitude and extrapolation of these results are supported by the large and moderate effect sizes in all OHIP-14 domains over the evaluated time. However, the patients showed a worse OHRQoL in the psychological discomfort domain when evaluated before root canal treatment. Only this domain showed a better response change after seven days of the procedure (responsiveness perfect), being clinically relevant and reflecting a real effect on this item.

However, future research focused on comparing the root canal treatment with different pulp and periapical diagnoses should be carried out to improve the power of this evidence, mainly focusing on patients’ needs and preferences, allowing the choice of the best treatment, always considering the risks and benefits. The objective of analyzing a treatment with OHRQoL instruments can be very useful in this clinical decision-making process, as it is an important complement to clinical data.^1,3^

## Conclusions

With large and moderate effect sizes, root canal treatment improved OHRQoL scores in all domains assessed by OHIP-14. In addition, female gender, mixed race and afrodescendant participants, maxillary teeth, and extensive crown destruction negatively influenced the OHRQoL of patients pre-RCT.

## Data Availability

The authors declare that all data generated or analyzed during this study are included in this published article.
